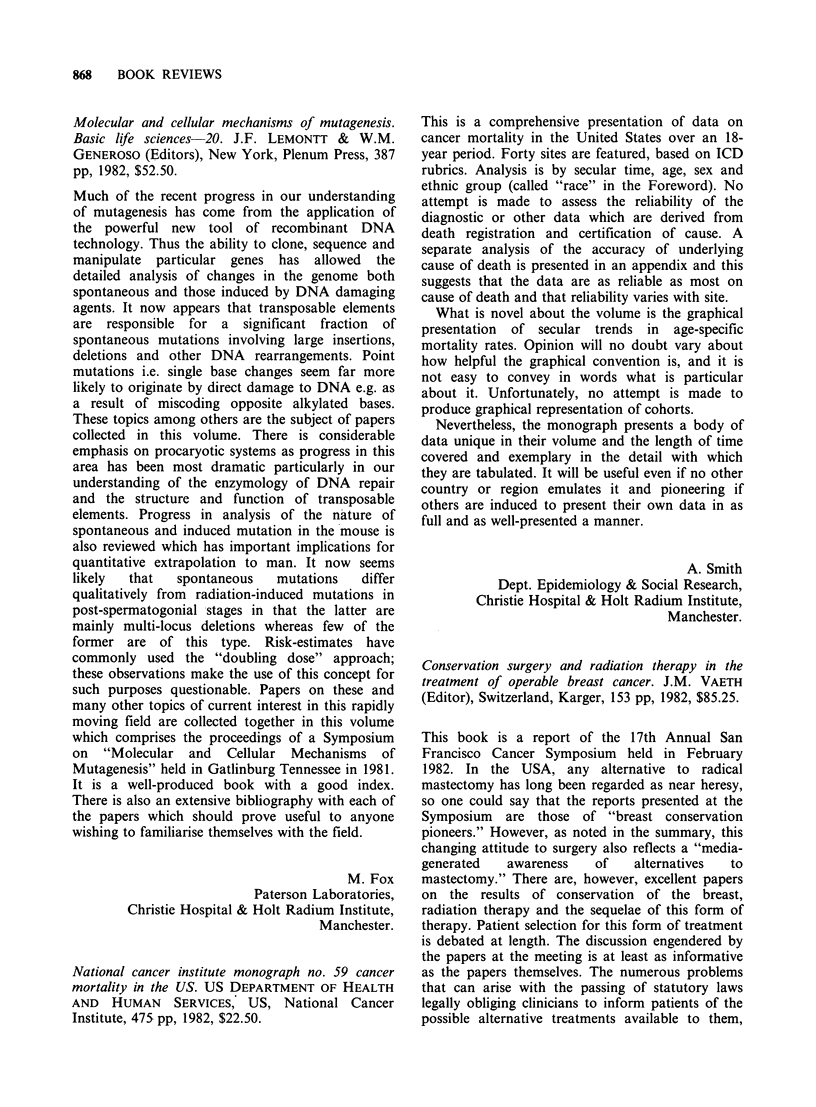# National cancer institute monograph no. 59 cancer mortality in the US

**Published:** 1983-06

**Authors:** A. Smith


Christie Hospital & Holt Radium Institute,

Manchester.

*National cancer institute monograph no. 59 cancer mortality in the US.* US DEPARTMENT OF HEALTH AND HUMAN SERVICES, US, National Cancer Institute, 475 pp, 1982, $22.50.

This is a comprehensive presentation of data on cancer mortality in the United States over an 18-year period. Forty sites are featured, based on ICD rubrics. Analysis is by secular time, age, sex and ethnic group (called "race" in the Foreword). No attempt is made to assess the reliability of the diagnostic or other data which are derived from death registration and certification of cause. A separate analysis of the accuracy of underlying cause of death is presented in an appendix and this suggests that the data are as reliable as most on cause of death and that reliability varies with site.

What is novel about the volume is the graphical presentation of secular trends in age-specific mortality rates. Opinion will no doubt vary about how helpful the graphical convention is, and it is not easy to convey in words what is particular about it. Unfortunately, no attempt is made to produce graphical representation of cohorts.

Nevertheless, the monograph presents a body of data unique in their volume and the length of time covered and exemplary in the detail with which they are tabulated. It will be useful even if no other country or region emulates it and pioneering if others are induced to present their own data in as full and as well-presented a manner.

A. Smith
Dept. Epidemiology & Social Research,
Christie Hospital & Holt Radium Institute,
Manchester.